# Revealing the Existence of Diverse Strategies for Phosphorus Solubilization and Acquisition in Plant-Growth Promoting *Streptomyces misionensis* SwB1

**DOI:** 10.3390/microorganisms13020378

**Published:** 2025-02-09

**Authors:** Yunzhu Chen, Zhuangzhuang Gao, Yan Yang, Qiang Liu, Lijuan Jiang, Jingzhen Chen, Xiao Zhou, Luhong Zhang, Yuena Ji, Jia Tu, Zhihong Xiao, Peiwang Li, Changzhu Li

**Affiliations:** 1 State Key Laboratory of Utilization of Woody Oil Resource, Hunan Academy of Forestry, Changsha 410004, China; cyzcarol@163.com (Y.C.); gaozz1129@foxmail.com (Z.G.); yangyanzupei@126.com (Y.Y.); chenjingzhen621@sina.com (J.C.); zx924647234@163.com (X.Z.); 20210100019@csuft.edu.cn (L.Z.); jyn13875854782@163.com (Y.J.); tujiashanito@gmail.com (J.T.); xiaozhihong@hnlky.cn (Z.X.); 2 College of Life Science and Technology, Central South University of Forestry and Technology, 498 South Shaoshan Road, Changsha 410004, China; liu.qiangcs@163.com (Q.L.); znljiang2542@163.com (L.J.)

**Keywords:** phosphate solubilizer, phosphorus mobilization mechanism, comparative genomics, *Cornus wilsoniana*, microbial fertilizer

## Abstract

Phosphorus deficiency poses a significant challenge to plant growth and development, particularly in red soil. To alleviate this limitation, phosphorus-solubilizing bacteria (PSB) play a crucial role by converting insoluble phosphates present in the soil into soluble forms that are accessible to plants. *Cornus wilsoniana* Wangerin is a representative oil crop cultivated in red soil, holding a prominent position within China’s forestry economic system. Consequently, it is essential to develop highly stable microbial phosphorus enhancement strategies to manage agricultural phosphorus in red soil regions, thereby maintaining the available phosphorus content necessary for the production of *C. wilsoniana*. In this study, the application of *Streptomyces misionensis* SwB1 bacterial suspension to the rhizosphere of *C. wilsoniana* significantly increased the content of various phosphorus fractions (H_2_O-P, NaHCO_3_-P, NaOH-P, HCl-P) in red soil, with NaHCO_3_-P content increasing by 4.97 times and NaOH-P content by 3.87 times. Additionally, the genome of *S. misionensis* SwB1 contains 25 phosphorus-solubilizing genes, 13 nitrogen-fixing genes, 17 siderophore production genes, and 11 indole-3-acetic acid (IAA) production genes, indicating its potential for enhancing nutrient availability. Comparative genomic analysis of 15 strains belonging to five species of *Streptomyces* revealed that *S. misionensis* SwB1 possesses an extensive genetic repertoire and complete gene clusters associated with phosphorus solubilization. Furthermore, five phosphorus solubilization pathways of *S. misionensis* SwB1 were summarized: the Pst system, Pit system, siderophore transport, phosphatase synthesis, and organic acid synthesis. Ultimately, the inoculation of *S. misionensis* SwB1 significantly enhanced the growth and biomass accumulation of *C. wilsoniana* at the seedling stage, evidenced by an increase in fresh weight by 81.44%, a rise in net photosynthetic rate by 18.51%, and a surge in the number of root tips by 36.24%. Taken together, our findings support a sophisticated multi-pathway bacteria phosphorus solubilization approach and identified a highly efficient phosphorus-solubilizing strain, *S. misionensis* SwB1, which has the potential to become a microbial fertilizer.

## 1. Introduction

Nowadays, ensuring sufficient access to soil nutrients globally poses a significant challenge to the sustainability of modern agriculture [1]. Due to its crucial role in converting solar energy into food, fiber, and other plant products, phosphorus is the major nutrient that can limit plant growth despite its abundance in soils in both inorganic and organic forms [2]. In red soil, which covers approximately 22.7% of China’s land area, insoluble phosphorus predominates, posing challenges for absorption and accumulation by plants [3]. Until now, the excessive application of chemical fertilizers and pesticides has directly and indirectly compromised the soil environment and crop yields [4]. Only 5–25% of the applied phosphorus can be absorbed by plants, while the remainder remains in the soil in insoluble forms [5]. Converting insoluble phosphorus into available phosphorus by phosphorus-solubilizing bacteria (PSB) is an effective way to enhance phosphorus utilization efficiency, soil structure, and plant production [6]. In modern agriculture, there is an urgent need for the use of phosphorus-solubilizing bacteria as biofertilizers to enhance crop productivity and augment nutritional security [7].

Phosphorus-solubilizing bacteria utilize various mechanisms to dissolve insoluble phosphorus [8]. These mechanisms include (i) organic acids, which can reduce pH and dissolve insoluble inorganic phosphorus. Gluconic acid, in particular, demonstrates effectiveness in enhancing the solubility of insoluble inorganic phosphorus in acidic soil and mitigating aluminum toxicity to plants [9]. Glucose dehydrogenase (GDH) catalyzes the synthesis of gluconic acid, requiring the combination with pyrrole quinoline quinone (PQQ) to form an active holoenzyme [10]. (ii) Phosphatases, which mineralize organic phosphorus and enhance the availability of soil organic phosphorus [11]. Most bacteria possess at least one of three phosphatases (PhoA, PhoX, and PhoD) exhibiting broad substrate specificity [12]. (iii) The Pst transport system, which is a protein channel responsible for phosphorus transportation and consumes energy [13]. Typically operating in low phosphorus environments, this system efficiently binds and transports inorganic phosphorus into the cytoplasm [14]. The phosphate-specific transport (Pst) system consists of peripheral-binding proteins (PstS), transmembrane proteins (PstA and PstC), and ATP-binding proteins (PstB), and regulatory protein (PhoU) [15].

*Cornus wilsoniana* Wangerin is a novel woody oil plant. The fruit oil of *C. wilsoniana* possesses a potent hypolipidemic effect, which is attributed to its high concentration of polyunsaturated fatty acids, particularly linoleic acid and γ-linolenic acid [16]. In China, *C. wilsoniana* is commonly cultivated in mountainous regions with red soil characterized by high iron content and low phosphorus availability. However, the production and quality of its fruit oil are significantly constrained by the phosphorus fixation by iron and other elements [17,18]. Thus, efficient and sustainable approaches to provide available phosphorus to *C. wilsoniana* are of great concern.

Our previous study identified a strain of *Streptomyces misionensis* SwB1, which has a highly efficient phosphorus solubilization ability [19]. However, the detailed mechanism of phosphorus solubilization mediated by *Streptomyces misionensis* SwB1 has not yet been completely elucidated. In the present study, genome sequencing and comparative genomic analysis were used to analyze the genetic composition characteristics and phosphorus solubilization mechanism of *Streptomyces misionensis* SwB1. Mechanistically, *Streptomyces misionensis* SwB1 solubilizes phosphorus by secreting organic acid, phosphatase, siderophore, and Pst phosphorus transport system. In addition, *Streptomyces misionensis* SwB1 has a variety of plant growth-promoting genes, which can effectively promote the biomass accumulation, photosynthesis, and root development of *C. wilsoniana*. Our findings support a basis for the research, development, and later application of this strain as a microbial fertilizer.

## 2. Materials and Methods

### 2.1. Plant Materials and Microorganisms

*Cornus wilsoniana* cultivar “Xianglin G1” saplings were provided by the *C. wilsoniana* germplasm repository of the Economic Forest Research Institute of Hunan Academy of Forestry (113°00′ E, 28°11′ N in Changsha, China), where the glasshouse experiment was conducted.

The *Streptomyces misionensis* SwB1 was grown on the International Streptomyces Program (ISP)-2 medium(MDBio, Qingdao, China) [20]. The matured spores were scraped off the colonies and stored in sterile glycerol at −80 °C.

### 2.2. Whole Genome Sequencing

The cryovial containing *S. misionensis* SwB1 is carefully removed from the −80 °C freezer and promptly thawed in a 37 °C water bath. Subsequently, in a strictly aseptic environment, the thawed bacterial suspension is meticulously transferred into pre-sterilized ISP-2 medium, and a sterile pipette is used to gently mix the suspension to ensure it is uniformly distributed throughout the ISP-2 medium. The conical flask is then carefully placed in a pre-calibrated incubator shaker set at 28 °C and 180 rpm to facilitate the efficient recovery and robust growth of the bacteria. After the *S. misionensis* SwB1 bacteria have been cultured to an appropriate density, the bacterial suspension is centrifuged at room temperature at 14,000× *g* for 5 min. The mycelium was then collected after discarding the medium, and DNA extraction and sequencing were performed. The sequencing was conducted by Frasergen, located at Wuhan, China. The PacBio Sequel II platform was employed for sequencing, followed by data processing using SMRTLINK 11.0.0 software [21,22]. After filtering low-quality and unknown sequences, the *S. misionensis* SwB1 genome was assembled using flye-2.9 and corrected by pilon-1.24 software based on paired reads [23,24].

For the strain *S. misionensis* SwB1 has been deposited in the China Center for Type Culture Collection under the accession number CCTCC NO. M 2023024. Furthermore, the genome sequence of *S. misionensis* SwB1 has been submitted to the NCBI database (GenBank ID: SAMN43297603).

Functional annotation was completed by aligning sequencing reads from the genome with the primary database. Essential databases used for *S. misionensis* SwB1 genome annotation include Non-Redundant Protein Sequence (NR), Clusters of Orthologous Groups (COG), Gene Ontology (GO), SwissProt, and Kyoto Encyclopedia of Genes and Genomes (KEGG) databases [25,26,27]. Genes involved in soil microbial plant growth-promoting function were searched in the KEGG database (https://www.genome.jp/kegg/mapper/ (accessed on 18 May 2024)).

### 2.3. Characterization of Growth-Promoting Ability

To investigate the plant growth-promoting properties of *S. misionensis* SwB1, we employed a variety of specialized growth media that target different mechanisms of plant growth promotion. These media were designed to assess the strain’s ability to solubilize inorganic and organic phosphorus, produce siderophores, synthesize indole-3-acetic acid (IAA), and promote growth through other means. All chemicals used for the preparation of these media were of analytical grade (MDBio, Qingdao, China). Below, we detail the composition of each medium and the observations made following the cultivation of *S. misionensis* SwB1.

The mycelia of *S. misionensis* SwB1 were added to media supplemented with inorganic phosphorus medium and organic phosphorus medium. After 7 days of culture, the formation of a transparent circle around the colony indicated phosphorus solubilization. The inorganic phosphorus medium included glucose 10 g, (NH_4_)_2_SO_4_ 0.5 g, yeast extract 0.5 g, NaCl 0.3 g, KCl 0.3 g, MgSO_4_ 0.3 g, MnSO_4_ 0.03 g, Ca_3_(PO_4_)_2_ 5 g, AGAR 15.0 g, deionized water 1 L, and pH = 7.0–7.5. The organic phosphorus medium included glucose 10 g, (NH_4_)_2_SO_4_ 0.5 g, yeast extract 0.5 g, NaCl 0.3 g, KCl 0.3 g, MgSO_4_ 0.3 g, MnSO_4_ 0.03 g, Ca_3_(PO_4_)_2_ 1 g, lecithin 0.2 g, AGAR 15.0 g, deionized water 1 L, and pH = 7.0–7.5.

The CAS detection medium for assessing siderophore production was composed of Chromium azurazine S (CAS) 0.0605 g, sodium dihydrogen phosphate dihydrate 0.29525 g, sodium hydrogen phosphate dodecahydrate 1.2135 g, HDTMA 0.0729 g, NaCl 0.0625 g, FeCl_3_·6H_2_O 0.002645 g, AGAR 9.0 g, NH_4_Cl 0.125 g, KH_2_PO_4_ 0.0375 g, deionized water 1 L, and pH adjusted to 6.7–6.9. After 7 days of culture, the formation of an orange-red zone around the colony signified siderophore production [28].

For the IAA production assay, the IAA assay medium included L-tryptophan 2.0 g, peptone 10.0 g, beef paste 3.0 g, NaCl 5.0 g, deionized water 1 L, and pH = 7.0–7.4 [29]. After 7 days of cultivation, the culture filtrate was obtained by centrifugation at 4000 rpm for 20 min, 1 mL of the supernatant of the centrifuged medium was taken, and 1 mL of Salkowski reagent was added. After the reaction in the dark for 30 min, the supernatant turned pink, indicating that the strain could produce IAA.

Lastly, the Ashby medium was used to assess the overall plant growth-promoting potential and was composed of KH_2_PO_4_ 0.2 g, NaCl 0.2 g, MgSO_4_ 0.2 g, K_2_SO_4_ 0.1 g, CaCO_3_ 5.0 g, glucose 10.0 g, AGAR 15.0 g, deionized water 1 L, and pH = 7.2–7.6, 25 °C. The normal growth of the strain after 7 days of culture indicated that the strain had the ability to fix nitrogen [30].

### 2.4. Comparative Genomic Analysis

Genomic data for 14 *Streptomyces* strains across 5 species were downloaded from NCBI and annotated using Prokka 1.12 [31]. Homology analysis of the genome was performed using OrthoFinder 2.5.2 [32], followed by drawing a petal map in R and plotting the gene clusters with ChiPlot (https://www.chiplot.online/ (accessed on 22 May 2024)). Evolutionary analysis was performed using MEGA 11 (developed by the Molecular Evolutionary Genetics Analysis Team). The Average Nucleotide Identity (ANI) of each strain was calculated by CompareM 0.1.2 (developed by the DOE Joint Genome Institute).

### 2.5. Pot Assay

#### 2.5.1. Glasshouse Experimental Design

Spontaneous germination of *C. wilsoniana* saplings with uniform height was transplanted to 2.5 L plastic pots with one sapling per pot under glasshouse conditions. The substrate was sterilized with red soil. Four treatments were set up: red soil + water (CK_0_), red soil + *S. misionensis* SwB1 (SwB1_0_), red soil + water + seedlings (CK), red soil + *S. misionensis* SwB1 + seedlings (SwB1). Fifty milliliters of spore suspension (107 spore mL^−1^) of *S. misionensis* SwB1 was applied to the base of each sapling (CK, SwB1), and the same volume of water was added to the control. The plants were grown in a greenhouse at 27 °C under 16 h light/8 h dark photoperiod, and relative humidity of 50–80%. All plants were regularly watered without any fertilizers. Each treatment had 10 replicate samplings, and all pots were completely randomized. After 60 days of growth, all plants were harvested.

#### 2.5.2. Determination of Soil Phosphorus Content

Starting from the base of the seedling, carefully dig with a small spade to expose the roots. Gently move away the large soil clumps around the roots, taking care not to disturb the root growth area. Lightly remove the large soil particles attached to the roots. Use a soft-bristled brush to gently brush off the fine soil particles adhering to the roots; these particles are the rhizosphere soil samples. These rhizosphere soils will be used to determine soil phosphorus content, including H_2_O-P, NaHCO_3_-P, NaOH-P, and HCl-P content were determined. The phosphorus was sequentially extracted by H_2_O, NaHCO_3_, NaOH, and HCl, and the phosphorus content was determined by molybdenum antimony resistance colorimetry [33]. Soil samples were collected every five days and measured. The measurement was performed in triplicates.

#### 2.5.3. Plant Growth Parameter Analyses

The height of the seedling and ground-level stem diameters were measured. The whole plant was weighed for fresh biomass and then dried at 60 °C until constant weight for dry biomass determinations. Each treatment was performed with ten plants.

Three healthy leaves in the upper part of the saplings were selected for the determination of photosynthetic parameters. The net photosynthetic rate, intercellular CO_2_ concentration, and stomatal conductance were measured using a portable photosynthesis system Li-6400 [34] at 60% RH, 25 °C air temperature and 400 μmol mol^−1^ CO_2_. All measurements were carried out in the morning from 9:00 a.m. to 11:00 a.m. [35]. Each treatment was performed with ten plants.

Root morphological parameters, including total root length, root area, root volume, and the number of tips, were measured using a root automatism scanner (Epson Twain Pro 2.10, Purchased from Wseen, Hangzhou, China) coupled with WinRHIZO™2000 Professional Edition program software. Each treatment was performed with ten plants.

### 2.6. Statistical Analysis

For the pot assay, data are mean values of independent experimental repetitions. Depending on the experiments, differences among treatments were reanalyzed by unpaired *t*-test or one-way ANOVA, followed by Tukey’s multiple range, using GraphPad Prism 9.5.0 and taking *p* ≤ 0.0001, *p* ≤ 0.001, *p* ≤ 0.01 or *p* ≤ 0.05, based on unpaired experimental design, as significant.

## 3. Results and Discussion

### 3.1. Effects of S. misionensis SwB1 on Phosphorus-Solubilizing in Red Soil

In a previous study, we isolated a total of 55 culturable phosphorus-solubilizing bacteria from the rhizosphere soil of *C. wilsoniana*. Among these isolates, we identified a strain of *S. misionensis* SwB1, which exhibited the highest efficiency in phosphorus solubilization and demonstrated significant potential to enhance plant growth [19].

**Figure 1:**
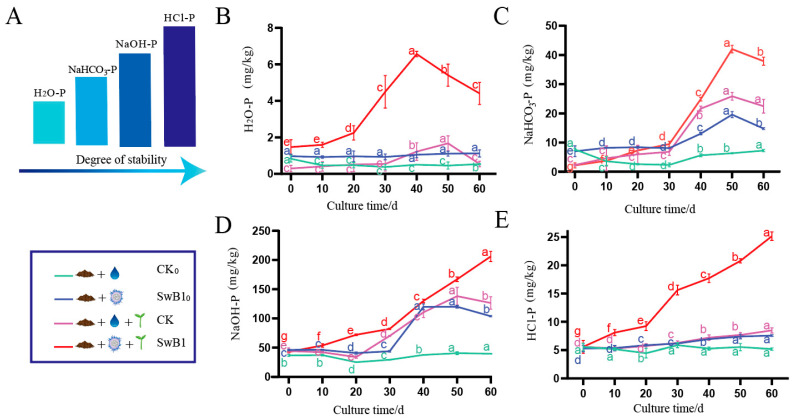
Effect of *S. misionensis* SwB1 on phosphorus content levels in red soil. (**A**) Phosphorus solubility schematic: HCl-P, NaOH-P, NaHCO_3_-P, and H_2_O-P, where darker colors indicate lower solubility. (**B**–**E**) Changes in H_2_O-P, NaHCO_3_-P, NaOH-P, and HCl-P content in soil samples under different treatments. Treatments included: red soil + water (CK_0_), red soil + SwB1 (SwB1_0_), red soil + water + seedlings (CK), and red soil + SwB1 + seedlings (SwB1), with three replicates per treatment. The letter markers indicate the significant differences determined by the ANOVA post hoc Tukey’s test, where the same letter denotes no significant difference (*p* > 0.05), and different letters indicate a significant difference (*p* ≤ 0.05). The error bars represent the standard error.

According to the Hedley phosphorus classification, inorganic phosphorus components can be categorized into four distinct categories: active inorganic phosphorus (H_2_O-P, NaHCO_3_-P), medium stable inorganic phosphorus (NaOH-P), stable inorganic phosphorus (HCl-P), and residual phosphorus (Figure 1A) [33]. H_2_O-P represents phosphorus in its ionic form within the soil, while NaHCO_3_-P refers to phosphorus that is adsorbed onto the soil surface and can be readily absorbed and utilized by plants. NaOH-P, on the other hand, is strongly attached to the soil surface through chemisorption. HCl-P is a solid phosphorus compound often coated with an iron and aluminum oxide film for closed storage [36,37].

To better understand the ability of *S. misionensis* SwB1 to solubilize various forms of phosphorus in red soil, this study measured the change in different levels of phosphorus (H_2_O-P, NaHCO_3_-P, NaOH-P, HCl-P) in red soils subjected to various treatments (Figure 1). After 60 days of incubation, the H_2_O-P content varied among the four treatment groups. The SwB1 group exhibited the highest increase at 2.98 times, followed by the CK group at 19.86%, and the SwB1_0_ group at 15.82%. In contrast, the CK_0_ group experienced a decrease of 44.35% (Figure 1B). Similarly, the NaHCO_3_-P content also showed varying changes; the SwB1 group experienced the highest increase at 4.97 times, followed by the CK group with an increase of 2.75 times, and the SwB1_0_ group with an increase of 1.11 times. The CK_0_ group decreased again, this time by 4.86% (Figure 1C). The NaOH-P content demonstrated significant changes as well, increasing by 3.87 times in the SwB1 group, 1.82 times in the CK group, and 1.38 times in the SwB1_0_ group, while the CK_0_ group saw a modest increase of 6.64% (Figure 1D). The HCl-P content also changed, with an increase of 3.51 times in the SwB1 group, 51.11% in the CK group, 43.26% in the SwB1_0_ group and increased by 6.78% in CK_0_ group (Figure 1E). Comparisons between the CK_0_ and SwB1_0_ treatment groups revealed that *S. misionensis* SwB1 significantly enhanced phosphorus content across all levels, especially in the NaHCO_3_-P and NaOH-P fractions. This indicates that the strain possesses the ability to alleviate the low phosphorus dilemma in red soil. Furthermore, comparisons between the CK and SwB1 groups demonstrated that the interaction between *C. wilsoniana* saplings and *S. misionensis* SwB1 strains facilitated the dissolution of insoluble phosphorus in red soil. It is possible that *S. misionensis* SwB1 promotes the growth of *C. wilsoniana* by improving phosphorus solubilization, and the well-grown *C. wilsoniana* also provides a beneficial living environment for *S. misionensis* SwB1, establishing a mutually beneficial relationship in the environment. Surprisingly, across all treatments, the CK group contributed to a comparable or even higher level of phosphorus content at various levels than the SwB1_0_ group. This may be attributed to the fact that the root exudates of *C. wilsoniana* seedlings include a wide array of organic acids, phenolic compounds, hormones, and other secondary metabolites [38]. These substances can more effectively solubilize the insoluble phosphorus in the soil. In contrast, the phosphorus-solubilizing ability of phosphate-solubilizing bacteria typically relies on specific enzymes or metabolic pathways, which may not be as diverse as the secretions from *C. wilsoniana* seedlings [39].

### 3.2. Genomic Characteristics and Functional Annotation

**Figure 2:**
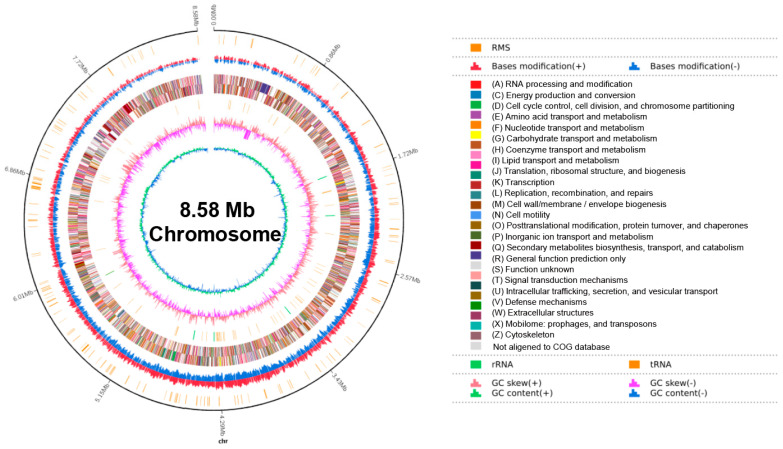
Circular representation of the *S. misionensis* SwB1 chromosome. The rings, labeled from the inside out, Ring 1, GC content (green for positive strand, blue for negative strand); Ring 2, GC skew (pink for positive strand, purple for negative strand); Ring 3, rRNA and tRNA (green and orange vertical lines, respectively); Rings 4 and 5, COG database annotations for positive and negative strands; Ring 6, base modifications (red for positive strand, blue for negative strand); Ring 7, enzymes associated with restriction-modification systems (orange vertical line).

**Figure 3:**
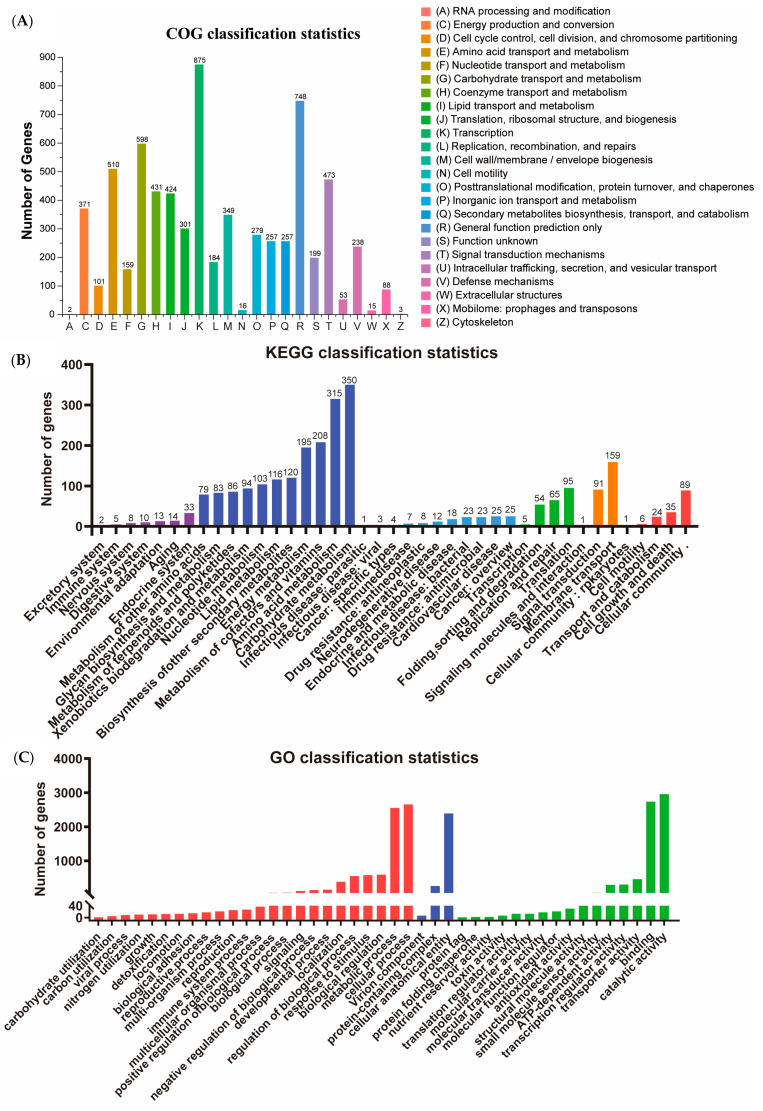
Annotated map of functional classification of *S. misionensis* SwB1 in whole genome. (**A**) COG functional annotation. (**B**) KEGG functional annotation. (**C**) GO function annotation. The *X*-axis represents GO function classifications, while the *Y*-axis indicates the number of genes associated with each classification. The numbers above each bar correspond to the total number of genes within each category.

The whole genome sequencing resulted in the assembly of a complete circular genome of *S. misionensis* SwB1(Figure 2), with a total length of 8,633,379 bp and a GC content of 72.45% (Table S1). The genome contains 8100 coding DNA sequence (CDS) genes, 82 tRNA genes, and 18 rRNA genes. Furthermore, a total of 12 gene islands, 62 CRISPR sequences, and 30 gene clusters were predicted within the genome. Notably, 72.89% of the genes were annotated in the COG database, and these genes were categorized into 26 functional classes (Figure 3A). The top five categories included transcription (875 genes), general function prediction only (748 genes), carbohydrate transport and metabolism (598 genes), amino acid transport and metabolism (510 genes), and signal transduction mechanisms (473 genes). Additionally, KEGG classification identified 2676 genes across six categories: cellular processes (CP), environmental information processing (EP), genetic information processing (GP), human diseases (HD), metabolism (MB), and organismal systems (OS) (Figure 3B).

A total of 4641 genes were annotated into three categories based on Gene Ontology (GO): Biological Process (BP), Cellular Component (CC), and Molecular Function (MF) (Figure 3C). The top five categories identified were catalytic activity (2958 genes), binding (2734 genes), cellular process (2657 genes), metabolic process (2553 genes), and cellular anatomical entity (2389 genes).

### 3.3. Evolutionary Analysis and Growth-Promoting Gene Analysis

**Figure 4:**
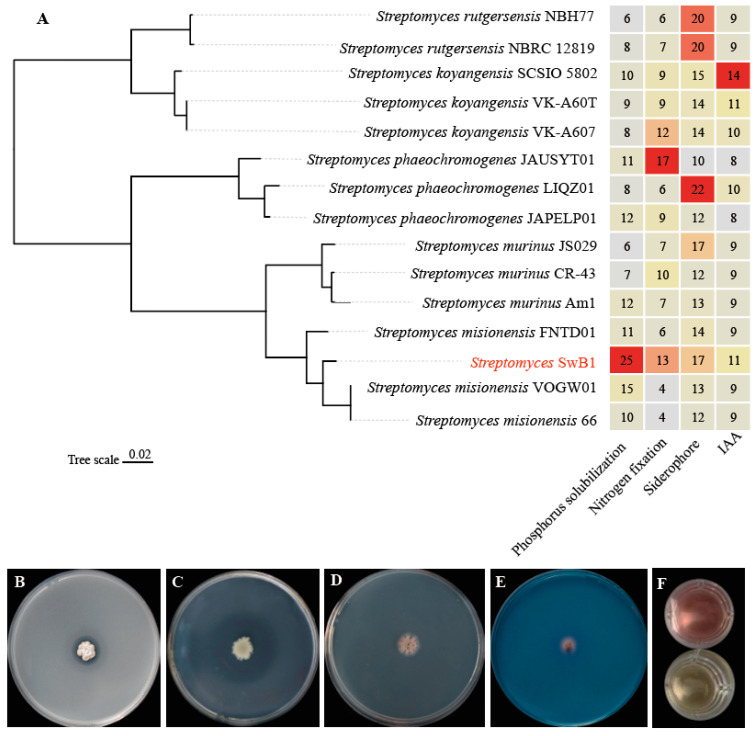
Characterization of plant growth-promoting the ability of *S. misionensis* SwB1. (**A**) phylogenetic relationships and growth-promoting genes among 15 *Streptomyces* strains. The cells in the heatmap are displayed in different colors, with hues closer to red indicating higher values at that position, and hues closer to gray indicating lower values. The values in the cells represent the number of growth-promoting genes in each strain. The text in red denotes the strains used in this study. (**B**) Inorganic phosphate solubilization. (**C**) Organic phosphate solubilization. (**D**) Siderophore production. (**E**) Nitrogen fixation. (**F**) IAA production.

The complete 16S rRNA sequence (1514 bp) of *S. misionensis* SwB1 was subjected to similarity-based searches in the NCBI database, and an evolutionary tree was constructed based on 16S rRNA sequences (Figure S1). The results showed that *S. misionensis* SwB1 is classified as *Streptomyces misionensis* with 100% cladistic confidence. To further elucidate the taxonomic position of *S. misionensis* SwB1, a phylogenetic tree was generated using Average Nucleotide Identity (ANI) analysis based on 15 complete genomes from other *Streptomyces* species (Figure 4A). As depicted in Figure 4A, *S. misionensis* SwB1, along with *Streptomyces misionensis* VOGW01, *Streptomyces misionensis* FNTD01, and *Streptomyces misionensis* 66, formed a monophyletic clade and was deeply nested within the tree, indicating a close evolutionary relationship. In contrast to *S. misionensis* SwB1, which was isolated from the rhizosphere soil, *Streptomyces misionensis* VOGW01, *Streptomyces misionensis* FNTD01, and *Streptomyces misionensis* 66 were isolated from the oasis and the ocean [40,41,42]. Given the differences in their respective niches, a comparative genome analysis of these strains may provide valuable insights into the specific adaptations and genomic evolution of *S. misionensis* SwB1.

To investigate the growth-promoting capabilities of *Streptomyces*, we conducted a comparative analysis in silico of the strain characteristics associated with phosphorus solubilization, nitrogen fixation, siderophore production, and indole-3-acetic acid (IAA) production among various *Streptomyces* strains (Figure 4A). The results revealed that *S. misionensis* SwB1 possessed the highest number of phosphorus-solubilizing genes (25), whereas *Streptomyces phaeochromogenes* JAUSYT01 exhibited the greatest number of nitrogen-fixing genes (17). Furthermore, *Streptomyces phaeochromogenes* LIQZ01 had the most siderophore production genes (22), and *Streptomyces koyangensis* SCSIO 5802 contained the highest number of IAA-producing genes (14). Moreover, the results indicated that the other *Streptomyces* strains possessed a greater number of genes related to nitrogen fixation, siderophore production, and IAA production in comparison to *S. misionensis* SwB1. In contrast, *S. misionensis* SwB1 demonstrated more advantageous characteristics regarding phosphorus solubilization. This result explained that *S. misionensis* SwB1 showed high efficiency in phosphorus solubilization in the red soil phosphorus transformation experiment (Figure 1).

The genomic annotation of *S. misionensis* SwB1 revealed the presence of genes associated with phosphorus solubilization, nitrogen fixation, siderophore production, and IAA production (Table 1 and Tables S2–S4). Consequently, this study investigated the role of *S. misionensis* SwB1 in promoting plant growth through experiments conducted on Petri plates. The capability to solubilize phosphorus and fix nitrogen may alter soil nutrient composition and structure, thereby enhancing the availability of soil nutrients [43]. In the phosphate solubilizing assay, the formation of a clear halo surrounding the colony indicates that *S. misionensis* SwB1 possesses the ability to solubilize both organic and inorganic phosphorus (Figure 4B,C). *S. misionensis* SwB1 was able to grow well in an Ashby medium, demonstrating the strain’s capability to fix nitrogen (Figure 4D). Furthermore, the secretion of siderophores and IAA has the potential to promote plant growth and enhance agronomic traits as well as stress resistance [44]. It also formed an orange halo on CAS agar medium, indicating siderophore production (Figure 4E). After 7 days of incubation with IAA detection medium inoculated with *S. misionensis* SwB1, the medium turned pink, whereas the control medium without *S. misionensis* SwB1 exhibited no color change, indicating the production of IAA (Figure 4F). In summary, the plant-promoting capabilities of *S. misionensis* SwB1, including phosphorus solubilization, nitrogen fixation, siderophores production, and IAA production, are consistent with the gene annotation results, thereby highlighting the potential of this strain as a microbial fertilizer. Previous studies have demonstrated that *Streptomyces misionensis* exhibits anticancer, insecticidal, and antibacterial activities [45,46,47,48]. However, this study represents the first investigation to highlight the plant growth-promoting properties, particularly its exceptional capability in phosphorus solubilization.

### 3.4. Comparative Genomics Analysis and Phosphorus Solubilization Gene Clusters

The mineralization of organic phosphorus and dissolution of inorganic phosphorus are critical mechanisms through which rhizosphere bacteria enhance soil AP content [49]. Twenty-five genes associated with phosphorus transport, phosphatase synthesis, and gluconic acid production were identified in the *S. misionensis* SwB1 genome (Table 1). Genes involved in phosphorus transport include *pstA*, *pstB*, *pstC*, *pstS*, *phoU*, and *pit*, which are part of the phosphate special transport system (Pst) and phosphate inorganic transport system (Pit) [50,51]. Among these, four paralogous genes are associated with phosphatase activity: *phoA*, *phoD*, *phoX*, and *phoE*. Additionally, gluconic acid synthesis includes the gluconate dehydrogenase gene (*gcd*) and cofactor synthesis genes: *pqqB*, *pqqC*, *pqqD*, and *pqqE* [52,53]. To investigate the plant growth-promoting genes of *S. misionensis* SwB1 further, a comparison was made with those of 15 Streptomyces strains from the NCBI database and previous studies. These 15 *Streptomyces* strains collectively contain 3195 core genes, accounting for 41.3% to 62.3% of their genome (Figure S2). The Pst phosphorus transport system, gluconic acid synthesis pathway, and phosphatase genes were analyzed across these 15 *Streptomyces* strains to explore their roles in phosphorus solubilization.

**Figure 5:**
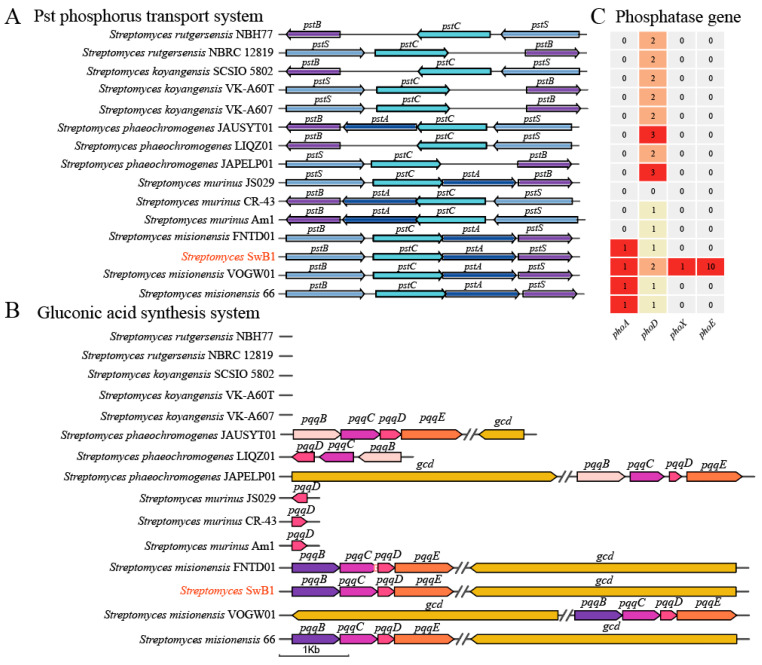
Comparison of phosphorolytic gene clusters and phosphatase genes among 15 *Streptomyces* strains. (**A**) Pst phosphorus transport system gene cluster. The length of the arrows corresponds to the length of the respective genes. The arrow’s direction indicates the gene’s orientation (5′ to 3′ from left to right). Each arrow is labeled with the corresponding gene name, and different genes are represented by different colors. The text in red denotes the strains used in this study. (**B**) Gluconic acid synthesis gene cluster. The text in red denotes the strains used in this study. (**C**) Heat map illustrating the types and quantities of phosphatase genes. The numbers in the heat map represent the quantity of genes. Darker shades correspond to a higher number of genes represented. The cells in the heatmap are displayed in different colors, with hues closer to red indicating higher values at that position, and hues closer to gray indicating lower values. The values in the cells represent the number of growth-promoting genes in each strain.

The Pst phosphorus transport system, comprising four proteins (PstA, PstB, PstC, and PstS), serves as the primary mechanism for phosphorus absorption in microorganisms under low-phosphorus conditions [54]. The Pst phosphorus transport system is anchored in the inner cell membrane by two multi-spanning proteins, PstC, and PstA, forming a transmembrane (TM) channel that facilitates phosphorus transport across the cytoplasmic membrane [55]. PstB is responsible for ATP hydrolysis, which energizes the TM channel, enabling it to function as a phosphorus transporter [56,57]. PstS is a peripheral binding protein with a high affinity for phosphorus, responsible for sensing and binding phosphorus in the periplasmic space [58]. In this study, fifteen strains of *Streptomyces*, including *Streptomyces murinus* and *Streptomyces misionensis*, possess a complete Pst phosphorus transport system (Figure 5A). Additionally, the homology and arrangement of genes in the Pst phosphorus transport system were entirely consistent among the four *Streptomyces misionensis* strains, including *S. misionensis* SwB1. However, some strains of *Streptomyces phaeochromogenes*, along with both *Streptomyces rutgersensis* and *Streptomyces koyangensis*, were deficient in the *pstA* gene. The *pstA* gene encodes an integral membrane protein with six transmembrane helices, and its disruption or loss decreases the protein’s affinity for membrane insertion, consequently impacting the Pst system, thereby reducing phosphorus transport and affecting the phosphorus uptake rate in microorganisms [59,60].

The gluconic acid synthesis system regulates the production of gluconate dehydrogenase and its coenzymes [61]. The *gcd* gene is responsible for regulating the production of glucose dehydrogenase in bacteria, and Pyrroloquinoline quinone (PQQ) serves as a coenzyme for glucose dehydrogenase. The synthesis of PQQ in bacteria requires the participation of the *pqqB*, *pqqC*, *pqqD*, and *pqqE* genes [62,63,64]. Strains of *Streptomyces misionensis* and *Streptomyces phaeochromogenes* possess a complete gluconic acid synthesis system. Interestingly, the number of genes in the gluconic acid synthesis system of the four *Streptomyces misionensis* strains was found to be completely consistent; however, the orientation of the PQQ gene in *Streptomyces misionensis* VOGW01 was found to be reversed. Strains of *Streptomyces phaeochromogenes* have a homologous PQQ synthesis gene, but the lengths of the *gcd* genes differed, with the *gcd* gene in *Streptomyces phaeochromogenes* LIQZ01 completely absent (Figure 5B), which may hinder gluconic acid synthesis for phosphorus dissolution [62]. Strains of *Streptomyces rutgersensis* and *Streptomyces koyangensis* may lack a complete gluconic acid synthesis system, which results in the loss of the ability to synthesize glucose dehydrogenase. This leads to the inability of the strain to convert to gluconic acid, thereby reducing its capacity to secrete organic acid-soluble phosphorus. These two bacterial strains had lost the *pstA* gene, which impaired their normal phosphorus uptake capability. Subsequently, they also lost the ability to synthesize gluconate, yet this additional loss did not impose further survival stress on them, possibly because their compromised phosphorus acquisition already placed them at a competitive disadvantage, rendering the impact of this further functional loss relatively limited.

Soil phosphatase is closely associated with soil phosphorus availability, functioning to hydrolyze phosphate groups of organophosphorus and promoting phosphorus absorption by plants [11]. The genes *phoA*, *phoD*, and *phoX* are the most common conserved phosphatase genes in bacteria, which hydrolyze phosphate monolipids to release phosphate ions [65]. This study compared the phosphatase genes of 15 *Streptomyces* strains, all of which contained *phoD* genes except *Streptomyces murinus* JS0295. Additionally, four strains of *Streptomyces misionensis* contained *phoA* genes. Notably, *S. misionensis* SwB1 possesses three common phosphatase genes—*phoD*, *phoX*, and *phoA*—along with 10 *phoE* genes, which have been annotated as a broadly specific phosphatase. This variety of phosphatase genes not only highlights the genetic diversity within *S. misionensis* SwB1 but also emphasizes the overall type and quantity of phosphorus-solubilizing genes present, which are crucial as they reflect the plant growth-promoting ability of microbials [29]. Furthermore, the abundance of *phoE* genes may significantly enhance the phosphorus solubilization ability of the *S. misionensis* SwB1 strain, which exhibits the most extensive variety of phosphorus solubilizing genes. This extensive gene repertoire and a complete gene cluster (Table 1) are essential for the strain’s remarkable capability to solubilize phosphorus effectively.

### 3.5. Effects of S. misionensis SwB1 Application on Physiological and Biochemical Regulation of C. wilsoniana

In recent years, microbial inoculation has emerged as a promising method for enhancing soil properties and increasing crop production [66].This study demonstrated that the *S. misionensis* SwB1 strain exhibits excellent phosphorus solubilization ability. The inoculation of *S. misionensis* SwB1 on the roots of *C. wilsoniana* significantly enhances phosphorus solubilization (Figure 1). Genome annotation identified numerous phosphorus solubilization genes, as well as nitrogen fixation genes, siderophore production genes, and IAA production genes in the *S. misionensis* SwB1 strain (Table 1). To further validate the plant growth-promoting ability of the *S. misionensis* SwB1 strain, a pot experiment was conducted using red soil as the substrate to assess its effect on the growth of *C. wilsoniana* seedlings.

**Figure 6:**
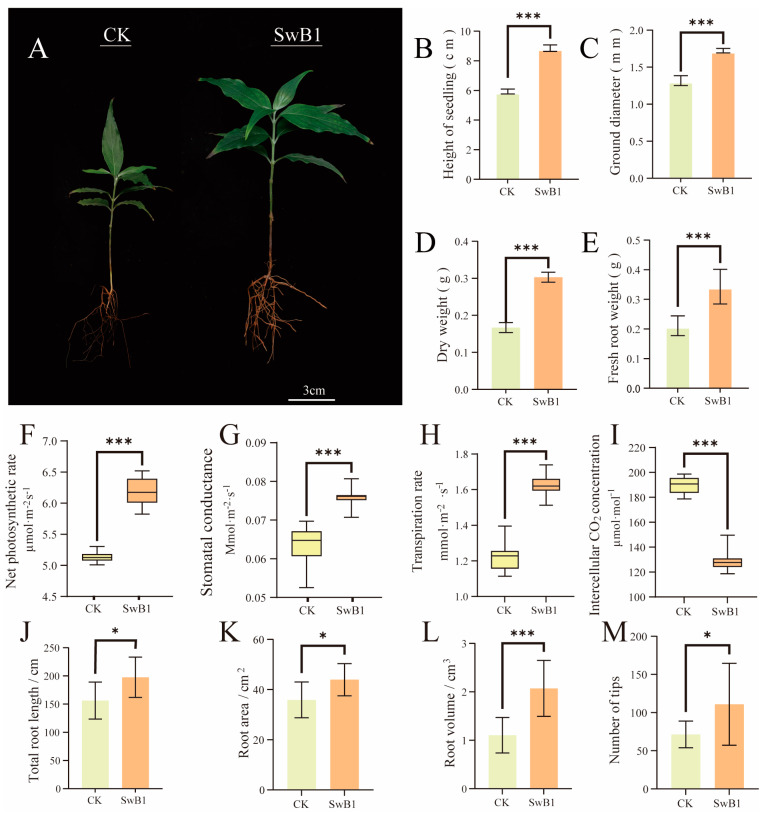
Effect of *S. misionensis* SwB1 on the morphological indices of *C. wilsoniana*. (**A**) Comparison of morphological characteristics of *C. wilsoniana* saplings. (**B**) Height of seedlings. (**C**) Ground diameter. (**D**) Dry weight. (**E**) Fresh weight. (**F**) Net photosynthetic rate. (**G**) Stomatal conductance. (**H**) Transpiration rate. (**I**) Intercellular CO_2_ concentration. (**J**) Total root length. (**K**) Root area. (**L**) Root volume. (**M**) The number of root tips. *, 0.01 < *p* ≤ 0.05; ***, *p* ≤ 0.001; the error bar represents the standard error (SE).

The SwB1 group caused a significant increase in seedling height (49.41%, *p* ≤ 0.05) and ground diameter (30.61%) compared to the CK group (Figure 6A–C). Additionally, *S. misionensis* SwB1 application significantly enhanced the biomass, with fresh weight increasing by 115% and dry weight by 81.44% compared to the CK group (Figure 6D,E). Overall, these results indicate that *S. misionensis* SwB1 promotes the growth and nutrient accumulation of *C. wilsoniana* seedlings.

*S. misionensis* SwB1 positively influenced both the leaf area and photosynthetic physiology of the seedlings. As the leaf area of *C. wilsoniana* increased, the net photosynthetic rate in the SwB1 group rose by 18.51% compared to the CK group (Figure 6F). Correspondingly, the leaf transpiration rate and stomatal conductance were significantly higher in the SwB1 group (*p* ≤ 0.05), with increases of 32.79% and 34.21%, respectively (Figure 6G,H). Additionally, the intercellular CO_2_ concentration in the leaves decreased by 34.21% compared to the CK group (*p* ≤ 0.05) (Figure 6I), which was consistent with the increase in the net photosynthetic rate [37]. These results demonstrate that *S. misionensis* SwB1 significantly enhances photosynthesis in *C. wilsoniana* leaves, leading to a greater accumulation of photosynthetic products and promoting overall plant growth.

The efficiency of nutrient absorption in plants is closely linked to root development, which enhances nutrient uptake and promotes growth. Scanning the root system of *C. wilsoniana* seedlings revealed that *S. misionensis* SwB1 promotes root development. Compared to the CK group, SwB1-treated seedlings exhibited a 26.28% increase in total root length (Figure 6J), with significant improvements in root area and volume—22.49% and 88.18% increases, respectively (Figure 6K,L). The number of root tips also increased significantly, with a 36.24% rise in the SwB1 group (Figure 6M). Therefore, *S. misionensis* SwB1 enhances the root system development of *C. wilsoniana*, induces root meristematic activity, and expands the contact area between roots and soil, thereby improving nutrient absorption and promoting the growth of the above-ground parts of the plant.

### 3.6. Proposing a High-Efficiency Dissolved Phosphorus Model for S. misionensis SwB1

**Figure 7:**
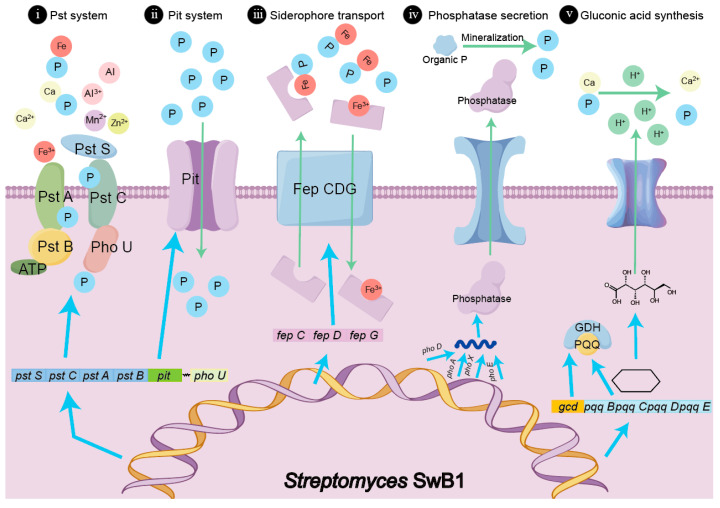
Illustrated schematic of the pivotal mechanism by which *S. misionensis* SwB1 facilitates phosphorus solubilization.

Different phosphorus-solubilizing bacteria use various mechanisms to solubilize phosphorus [67]. We have compiled a study on *S. misionensis* SwB1, which sheds light on its phosphorus solubilization pathway. Our findings indicate that this strain exhibits diverse modes of phosphorus enhancement, which include a Pst system, Pit system, siderophore transport system, phosphatase secretion system, and gluconic acid secretion system (Figure 7).

The genome of the *S. misionensis* SwB1 strain encompasses five distinct phosphorus solubilization systems: (i) The Pst system comprises genes for peripheral-binding proteins (PstS), transmembrane proteins (PstA and PstC), ATP-binding proteins (PstB), and regulatory proteins (PhoU), facilitating phosphate transport from iron, aluminum, and calcium phosphates under low-phosphorus conditions [68]. (ii) The *pit* gene encodes a rapid phosphorus transport system that operates optimally in high phosphate ion concentrations, allowing for cellular uptake through specialized channels [69]. (iii) Genes *fepC*, *fepD*, and *fepG* form the siderophore transport system, which is crucial for phosphorus dissolution in red soil; siderophores, which have a high affinity for iron, help prevent phosphorus immobilization by forming complexes with iron in iron phosphate [37,70]. (iv) Four phosphatase genes (*PhoA*, *PhoD*, *PhoX*, and *PhoE*) enhance phosphorus solubilization in red soil by converting organic phosphate into available phosphorus (AP), vital for plant growth [71]. (v) Gluconic acid, produced through the interaction of glucose dehydrogenase (GDH) and pyrroloquinoline quinone (PQQ), facilitates phosphorus dissolution [72,73]. The *gcd* gene catalyzes gluconic acid production, along with the genes synthesizing PQQ (pqqB, pqqC, pqqD, and pqqE) identified in the genome. The microbial release of gluconic acid lowers soil pH, enhances the breakdown of insoluble inorganic phosphorus, and increases available phosphorus content. The combined effects of these mechanisms enhance *S. misionensis* SwB1’s phosphorus solubilization capacity, improving phosphorus utilization by *C. wilsoniana* cultivated in red soil.

## 4. Conclusions

In this study, *Streptomyces misionensis* SwB1, isolated from *C. wilsoniana*, was shown to significantly increase phosphorus content in red soil through its symbiotic relationship with *C. wilsoniana*. Genome sequencing revealed that*S. misionensis* SwB1 contains numerous phosphorus solubilizing genes, as well as genes related to nitrogen fixation, siderophore production, and IAA production, which were confirmed through plate verification. Comparative genomic analysis showed that *S. misionensis* SwB1 possesses a remarkable capacity for phosphorus solubilization, attributable to its complete phosphorus transport and gluconic acid synthesis systems, along with a higher number of phosphatase genes. *S. misionensis* SwB1 significantly enhanced nutrient uptake by *C. wilsoniana*, thereby improving plant growth and productivity through beneficial effects on physiological and biochemical parameters. To harness this microbial-mediated phosphorus solubilization as a sustainable strategy for boosting plant productivity and soil health, further studies are needed to elucidate the role of microorganisms in the growth regulation network of plants.

## Figures and Tables

**Table 1:** Phosphorus-solubilizing genes of *S. misionensis* SwB1.

Gene Name	Gene Annotation	Functional Classification	Gene Number
*pstA*	2-amino-1-hydroxymethyl phosphonate dioxygenase	Phosphorus transport	1
*pstB*	Phosphinothricin acetyltransferase	Phosphorus transport	1
*pstC*	Phosphinothricin acetyltransferase	Phosphorus transport	1
*pstS*	N-acetylglucosamine PTS system EIICBA or EIICB component	Phosphorus transport	1
*phoU*	Phosphate-specific transport system accessory protein PhoU	Phosphorus transport	1
*pit*	N-acetylglucosamine PTS system EIICBA or EIICB component	Phosphorus transport	1
*phoA*	Alkaline phosphatase H	Phosphatase synthesis	1
*phoD*	Alkaline phosphatase D	Phosphatase synthesis	2
*phoX*	Secreted phosphatase, PhoX family	Phosphatase synthesis	1
*phoE*	Broad specificity phosphatase PhoE	Phosphatase synthesis	10
*gcd*	Glucose dehydrogenase, PQQ-dependent	Gluconic acid synthesis	1
*pqqB*	Pyrroloquinoline quinone biosynthesis protein B	Gluconic acid synthesis	1
*pqqC*	Pyrroloquinoline-quinone synthase	Gluconic acid synthesis	1
*pqqD*	Pyrroloquinoline quinone biosynthesis protein D	Gluconic acid synthesis	1
*pqqE*	PqqA peptide cyclase	Gluconic acid synthesis	1

## Data Availability

The data presented in this study are openly available inGenBank, reference number SAMN43297603.

## Supplementary Materials

The following supporting information can be downloaded at: https://www.mdpi.com/article/10.3390/microorganisms13020378/s1, Figure S1. Phylogenetic analysis of *S. misionensis* SwB1. The bold font is SwB1. The “ T ” in the upper right corner of the strain represents the model strain. The percentage at the branch indicates branch confidence. Figure S2. Petal map of the genome of 15 strains. The strains in red font are the strains isolated in this study. The middle number is the number of genes shared by all strains. Each petal represents a unique gene; Table S1. *S. misionensis SwB1* genomic characterization information. Table S2. Nitrogen fixation gene of *S. misionensis SwB1*. Table S3. Siderophore production gene of *S. misionensis SwB1*. Table S4. IAA-producing gene of *S. misionensis SwB1*.
