# Supplementary material for: Revealing the Existence of Diverse Strategies for Phosphorus Solubilization and Acquisition in Plant-Growth Promoting Streptomyces misionensis SwB1
Source: Microorganisms. 2025 Feb 9;13(2):378. doi: 10.3390/microorganisms13020378 (PMC11858620; doi:10.3390/microorganisms13020378)
Supplement: Supplementary file 1 [file microorganisms-13-00378-s001.zip › microorganisms-3448550-supplementary.pdf]

Supplemental Information

Whole-Genome Sequencing and Comparative Genomics Analysis Reveals the Mechanism of a Potential Phosphate Solubilizer from *Streptomyces misionensis* SwB1

Yunzhu Chen [a, 1], Zhuangzhuang Gao[a,b,1], Yan Yang[a], Qiang Liu[b], Lijuan Jiang[b], Jingzhen Chen[a], Xiao Zhou [a,b], Luhong Zhang [a,b], Yuena Ji[a], Jia Tu[a], Zhihong Xiao[a], Peiwang Li [a,*], Changzhu Li[a,*]

Author affiliations

[a] State Key Laboratory of Utilization of Woody Oil Resource, Hunan Academy of Forestry, Changsha, Hunan 410004, PR China

[b] College of Life Science and Technology, Central South University of Forestry and Technology, 498 South Shaoshan Road, Changsha, Hunan 410004, PR China

[1] Authors contributed equally to this work.

*Correspondence

Email: Peiwang Li, lindan523@163.com

Changzhu Li, lichangzhu2013@aliyun.com

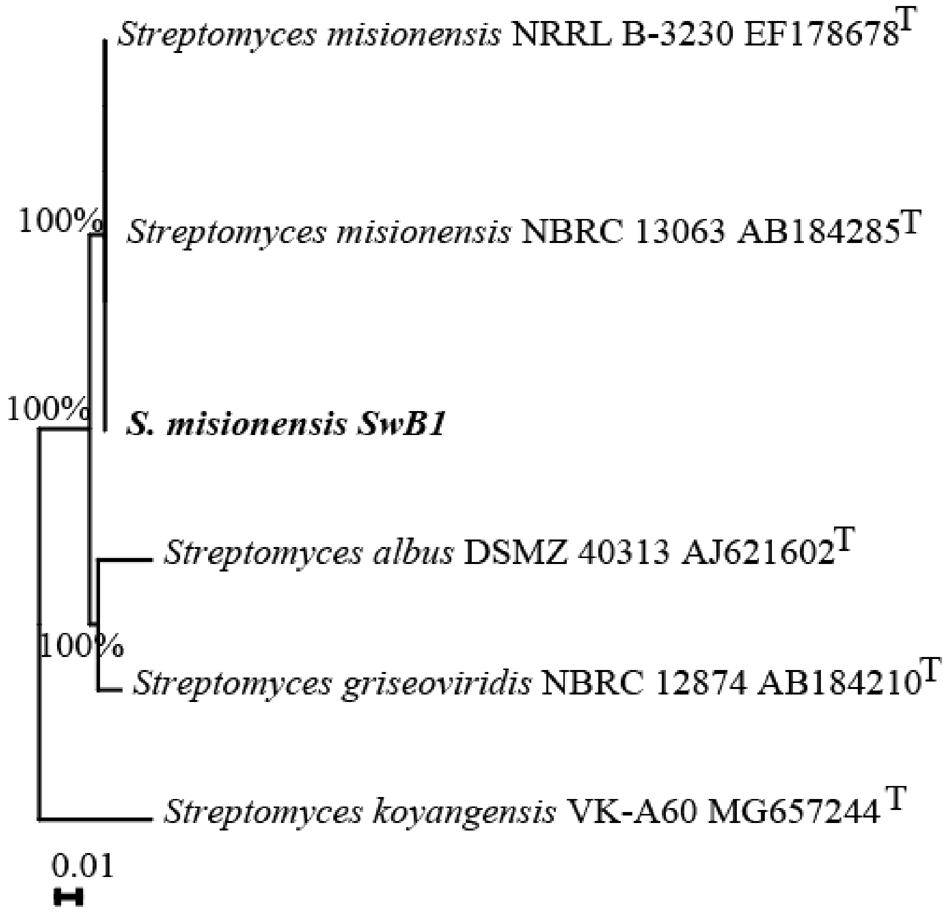

Figure S1 Phylogenetic analysis of *S. misionensis SwB1*. The bold font is SwB1. The " T " in the upper right corner of the strain represents the model strain. The percentage at the branch indicates branch confidence.

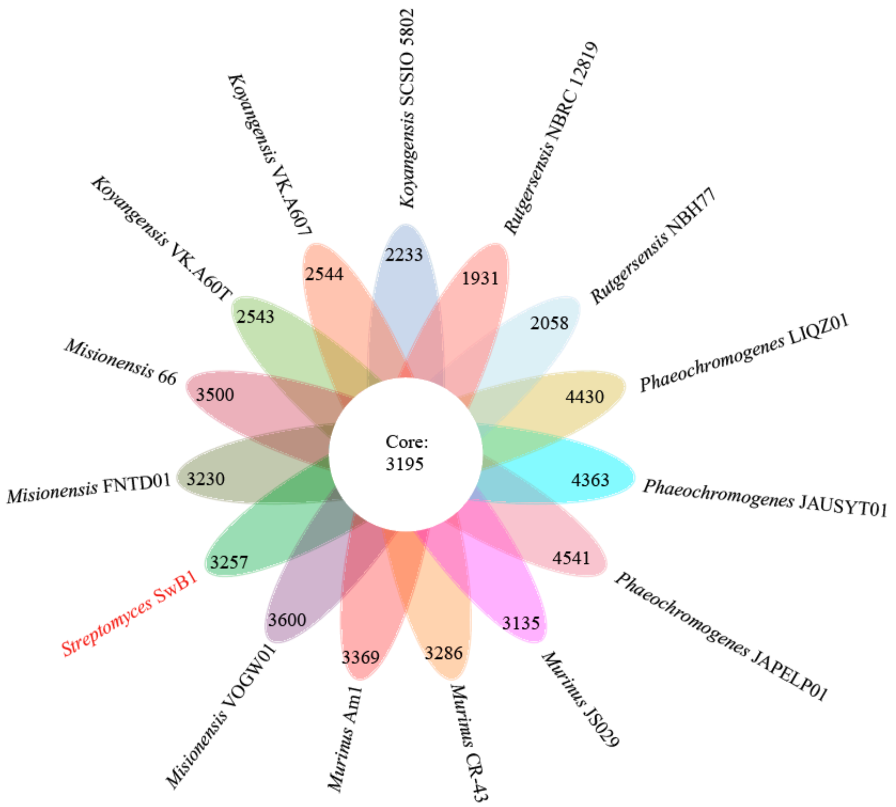

Figure S2 Petal map of the genome of 15 strains. The strains in red font are the strains isolated in this study. The middle number is the number of genes shared by all strains. Each petal represents a unique gene.

Table S1. *S. misionensis SwB1* genomic characterization information

Characteristics-SwB1	Value
Genome size (bp)	8,633,379
GC content (%)	72.45%
Topology	Circular
Chromosome size (bp)	8,579,758
Chromosome	1
Plasmid	1
tRNA	82
rRNA (5S,16S,23S)	18
Protein-coding genes (CDS)	8100
Genomic islands	12
CRISPR	62
Gene cluster	30
Genes assigned to NR	7623
Genes assigned to GO	4641
Genes assigned to KEGG	2676
Genes assigned to COG	5904
Genes assigned to Swiss-Prot	4765

Table S2 Nitrogen fixation gene of *S. misionensis SwB1*

Gene name	Gene annotation	Functional classification	Gene number
narI	nitrate reductase gamma subunit	Nitrogen metabolism	1
nmoA	nitronate monooxygenase	Nitrogen metabolism	1
nrfF	formate-dependent nitrite reductase complex subunit NrfF	Nitrogen metabolism	1
nasA	assimilatory nitrate reductase catalytic subunit	Nitrogen metabolism	1
nasB	assimilatory nitrate reductase electron transfer subunit	Nitrogen metabolism	1
narX	Nitrate reductase-like protein NarX	Nitrogen metabolism	1
nirB	nitrite reductase (NADH) large subunit	Nitrogen metabolism	1
nirD	nitrite reductase (NADH) small subunit	Nitrogen metabolism	1
pncB	nicotinate phosphoribosyltransferase	Nitrogen metabolism	1
glnB	Nitrogen regulatory protein PII	Nitrogen transport	1
narK	Nitrate/nitrite transporter NarK	Nitrogen transport	2
nifU	nitrogen fixation protein NifU and related proteins	Nitrogen fixation	1

Table S3 Siderophore production gene of *S. misionensis SwB1*

Gene name	Gene annotation	Functional classification	Gene number
fepC	iron complex transport system ATP-binding protein	Siderophore transport	1
fepG	iron complex transport system permease protein	Siderophore transport	1
fepD	iron complex transport system permease protein	Siderophore transport	1
viuB	NADPH-dependent ferric siderophore reductase (2)	Siderophore production	2
ybtE	yersiniabactin salicyl-AMP ligase	Siderophore production	1
entD	siderophore biosynthesis (2)	Siderophore production	2
fadA5	steroid 3-ketoacyl-CoA thiolase	Siderophore production	1
iucA	IucA/IucC family siderophore biosynthesis protein (5)	Siderophore production	5
iucC	IucA/IucC family siderophore biosynthesis protein	Siderophore production	1
iucD	IucA/IucC family siderophore biosynthesis protein	Siderophore production	1
paaY	siderophore binding protein	Siderophore production	1

Table S4 IAA-producing gene of *S. misionensis SwB1*

Gene name	Gene annotation	Functional classification	Gene number
iaaM	tryptophan 2-monooxygenase activity	IAA production	1
kynA	tryptophan 2,3-dioxygenase	IAA production	1
iacA	indole-3-acetate monooxygenase	IAA production	1
trpS	tryptophanyl-tRNA synthetase	IAA production	1
trpA	tryptophan synthase alpha chain (2)	IAA production	2
trpB	tryptophan synthase beta chain	IAA production	1
trpC	indole-3-glycerol phosphate synthase (2)	IAA production	2
trpE	anthranilate synthase component I	IAA production	1
trpF	phosphoribosyl anthranilate isomerase	IAA production	1
trpD	anthranilate phosphoribosyltransferase	IAA production	1
